# Simplifying CD4 Testing to Support Advanced HIV Disease Care

**DOI:** 10.1093/infdis/jiag438

**Published:** 2026-10-15

**Authors:** Ajay Rangaraj, Nathan Ford

**Affiliations:** 1 Department HIV, TB, Hepatitis, and STIs, World Health Organization, Geneva, Switzerland; 2 Centre for Integrated Data and Epidemiological Research, School of Public Health, University of Cape Town, Cape Town, Western Cape, South Africa

## Abstract

There were approximately 570 000 AIDS-related deaths in 2025—almost all among individuals with advanced human immunodeficiency virus (HIV) disease (AHD) [1]. AHD remains a persistent challenge despite widespread access to HIV testing and treatment. This is partly because people test late in their disease progression but increasingly because people who have tested and started treatment disengage from services, only to return to seek care once they are ill [2]. At least a third of individuals presenting to health care settings have AHD, a proportion that rises to over 40% in hospitals settings [3]. Household surveys in the sub-Saharan African region have found that approximately 9% of all people with HIV have AHD—an estimated 1.9 million people [4]. Thus, improvements in coverage of HIV testing and access to antiretroviral therapy sit uneasily alongside high rates of AHD and a slowing decline in AIDS-related deaths, presenting a conundrum to policymakers.

The World Health Organization (WHO) strongly recommends CD4 cell count testing as the preferred way to diagnose AHD [5]. However, country capacity to perform CD4 tests has declined in recent years following guideline recommendations that CD4 testing was no longer required to make decisions about starting antiretroviral therapy: one study noted declines from 79% to 13% in Southern Africa and from 85% to 31% in West Africa, with similar declines noted in other regions [6]. The pipeline of CD4 technologies has also reduced: in 2014, there were at least 6 commercially available point-of-care devices, with 4 more tests in development [7]; as of 2026, only a single true point-of-care device was available—the VISITECT CD4 Advanced Disease test [8].

A recent study published in The *Journal of Infectious Diseases* provides reassuring evidence supporting expanding access to CD4 testing [9]. The study found a very high level of agreement (95.5%) between the fingerprick and venous whole-blood samples for measuring CD4 cell count using a point-of-care CD4 test. As noted by the study investigators, fingerprick sampling requires little infrastructure, shortens wait times, and supports timely clinical decision making.

While additional training is needed, the test has been found to be generally feasible and acceptable to health workers [10]. There have been challenges in interpreting the test results, which is believed to be a contributing factor to the lower than expected specificity of the test. Studies conducted in Ethiopia, Indonesia, and Argentina have reported that retraining of health workers helped improve specificity [12, 13]. Finally, there are some limitations for performing this test at higher volumes, given it takes around 40 minutes per test, and the limited shelf life [8]. Implementing an instrument-free test presents many opportunities—lower operational costs, easier distribution and scale-up, no maintenance costs, and greater scope for task sharing [10]. The VISITECT CD4 Advanced Disease test has received WHO prequalification for use [8], but researchers have noted that the test has lower than expected specificity in field studies with retained test sensitivity [11]. Practically, this means that while very few people who have AHD will be missed, there will be an increase in the rate of false-positive diagnosis of AHD, which could lead to higher program costs.

Nevertheless, given the shrinking resources for HIV programs worldwide and the urgent need to screen and treat people with AHD, the instrument-free CD4 test supports a critical programmatic priority [14] alongside more centralized quantitative CD4 testing. Simplification of sample collection is a positive step in the right direction to improve test efficiency and allow for better task sharing practices to improve access to CD4 testing, and entry into the advanced HIV care pathway for those in need. Recent commitments by suppliers to maintain CD4 testing capacity are encouraging, although ongoing monitoring of product availability remains important: a recent survey of 8 countries in Africa by the Clinton Health Access Initiative and Unitaid reported a 35% reduction in CD4 tests run in 2025 compared to 2024 [15].

The 2025 WHO guideline reaffirms the importance of CD4 testing in reducing the burden of AHD and moving closer achieving the United Nations target of ending AIDS by 2030 [15], a target toward which many countries remain off track.
